# Réaction à El M’rabet FZ et al.: Métastase splénique d’un adénocarcinome colique - à propos d’un cas et revue de la littérature

**Published:** 2012-01-12

**Authors:** Youssef Alaoui Lamrani, Jaffal Mohammed, Imane Kamaoui, Siham Tizniti

**Affiliations:** 1 CHU Hassan II, Service de Radiologie, Fès, Maroc

**Keywords:** Métastase, rate, colon, carcinome

## Réaction

J’ai lu avec grand intérêt l’article de El M’rabet FZ. et al. portant sur un cas de métastase splénique d’un adénocarcinome colique [1]. La revue de la littérature est riche, toutefois, elle suscite quelques commentaires. Les formes symptomatiques des métastases spléniques, quoique moins fréquentes, auraient méritées d’être citées, car souvent révélatrices de complications sévères: douleurs de l’hypochondre gauche pouvant révéler une compression vasculaire, une thrombose de la veine splénique, ou une compression d’un organe de voisinage, un tableau d’abdomen aigu ou d’instabilité hémodynamique pouvant exprimer une rupture splénique. D’autres circonstances, moins spécifiques, peuvent susciter des investigations et être révélatrices de métastase splénique : fatigue, amaigrissement, état fébrile, ou signes d’anémie et de thrombopénie en rapport avec un hypersplénisme [2]. La décision de splénectomie prise dans ce rapport de cas, avait un but diagnostique et thérapeutique, et s’inscrivait dans un cadre programmé, et imposerait au préalable une vaccination efficace contre les bactéries encapsulées impliquées dans les infections graves chez les patients aspléniques (*Streptococcus pneumoniae*, *Haemophilus influenzae* de type b, et *Neisseria meningitidis*), cette vaccination aurait mérité d’être mentionnée [3].

Le diagnostic d’une métastase splénique isolée se base, essentiellement, sur des critères morphologiques ultrasonographiques ou tomodensitométriques au moment du diagnostic du cancer primitif ou lors du suivi radiologique régulier des patients atteints de cancers. Le recours à la TEP-FDG couplée au scanner serait d’un grand apport, en différenciant masse splénique bénigne et maligne, et en identifiant d’autres sites métastatiques hypermétaboliques insoupçonnées sur l’imagerie conventionnelle. L’imagerie de la métastase splénique pourrait également bénéficier de l’apport de l’IRM avec des produits de contraste super-paramagnétiques qui discernent facilement le tissu réticulo-endothélial du tissu tumoral. A partir des données de ces techniques, on peut voir l’indication d’une splénectomie éliminée, en détectant d’autres sites métastatiques ou en approchant la nature bénigne d’une lésion splénique mimant une métastase (hamartome et pseudo-tumeur inflammatoire) [3].

Le diagnostic de certitude d’une lésion splénique isolée est basé sur l’étude anatomo-pathologique, laquelle est réalisée classiquement, sur pièce de splénectomie évitant la ponction biopsie de la rate réputée pourvoyeuse de complications hémorragiques. Dans une étude récente, Cavanna L et al. ont démontré la réelle précision diagnostique de la ponction biopsie écho-guidée de la rate qui avoisine 98,1%, son caractère moins agressif, et son coût peu onéreux, comparativement à un geste chirurgical. Cette technique écho-guidée pourrait poser le diagnostic et éviter le recours à une splénectomie non nécessaire, voire néfaste chez un patient cancéreux, exemple fait d’une lésion splénique granulomateuse bénigne, ou d’un lymphome B primitif à grandes cellules pouvant donner le change avec une métastase [4].
